# Supplementary material for: Differential chromatin accessibility in peripheral blood mononuclear cells underlies COVID-19 disease severity prior to seroconversion
Source: Sci Rep. 2022 Jul 9;12:11714. doi: 10.1038/s41598-022-15668-8 (PMC9271053; doi:10.1038/s41598-022-15668-8)
Supplement: Supplementary file 1 — Supplementary Information 1. [file 41598_2022_15668_MOESM1_ESM.docx]

**SUPPLEMENTAL INFORMATION**

**Contents PDF**

**Supplemental Figure S1** Cohort Symptom Severity

**Supplemental Figure S2** Single cell RNA-seq/ATAC-seq

**Supplemental Table S1** Subjects serology and PCR results for bulk RNA-seq/ATAC-seq analysis

**Supplemental Table S2** Subjects serology and PCR results for single cell RNA-seq/ATAC-seq analysis

**Contents Excel**

**Supplemental Table S3** Gene symbols, gene names, fold change and p values adjusted for multiple hypothesis testing p <= 0.05 for RNA-seq of the four comparisons shown in Figure 1.

**Supplemental Table S4** Number of cells for each sample contributing to each scATAC-seq cluster.

**Supplemental Table S5** Number of cells for each sample contributing to each scATAC-seq cell type.

**Supplemental Table S6** Cell type marker expression in scATAC-seq data following integration with scRNA-seq

**Supplemental Table S7** Genes for transcription factors inferred from accessible chromatin using (A) footprint depth or (B) average per-cell gene expression (B) in IgG- COVID-19 subjects with mild or moderate symptoms

**Supplemental Table S8** Pathways used to calculate gene expression scores in monocyte subjects.

**Supplemental Table S9** Results of one-way analysis of variance and Tukey test for cell subset pathway scoring.

**Supplemental Figure S1**

**
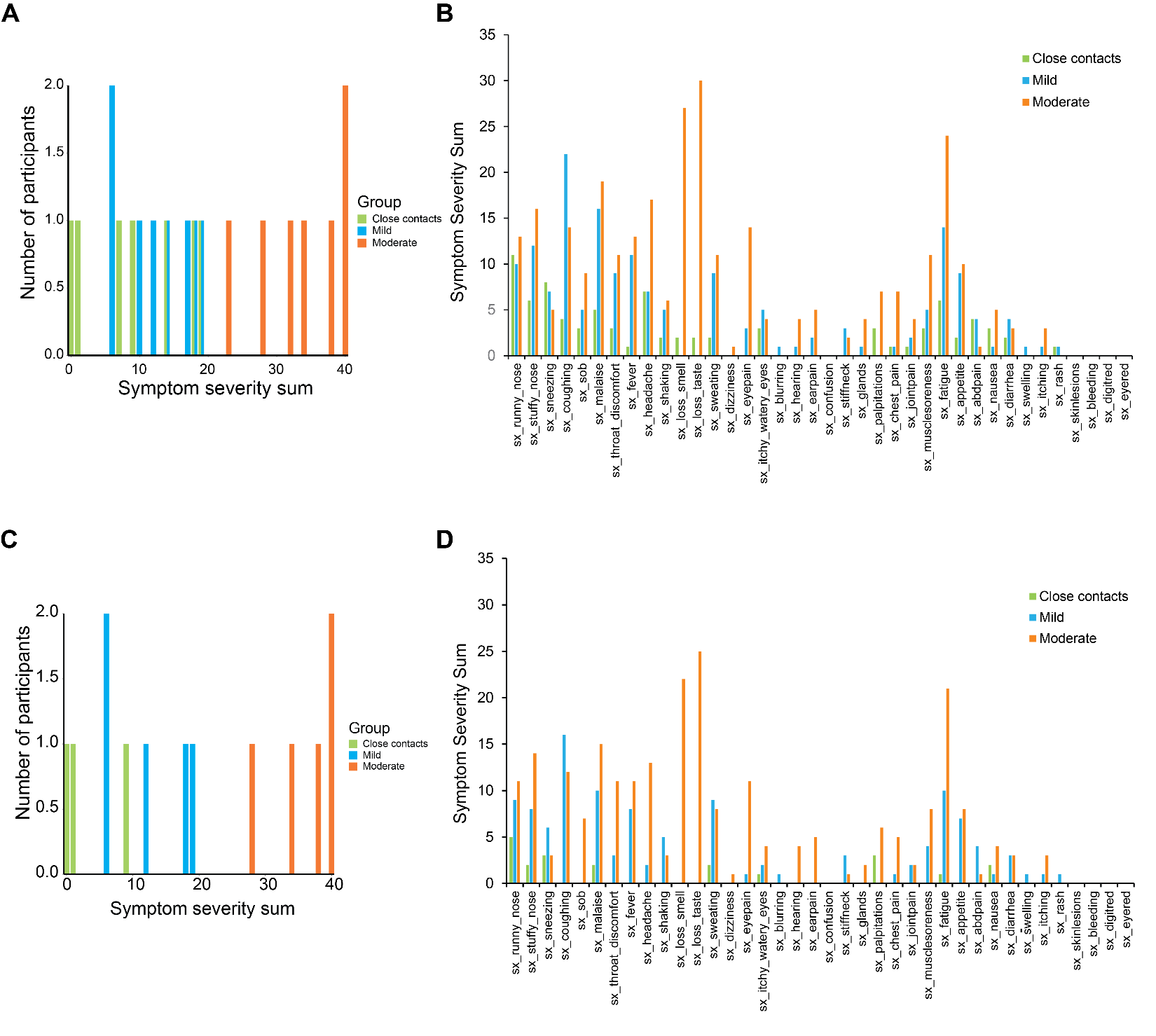
**

**Supplemental Figure S1. (A)** Symptom severity sums for the subjects profiled with bulk assays and **(B)** severity scores for each reported symptom for the subjects profiled with bulk assays**. (C)** Symptom severity sums for subjects profiled with single-cell assays and **(D)** severity scores for each reported symptom for the subjects profiled with single-cell assays. For both cohorts, the sum of severity scores for all reported symptoms are shown for close contacts (green), COVID-19 subjects with mild symptoms (blue), and with moderate symptoms (orange).

**Supplemental Figure S2**

**
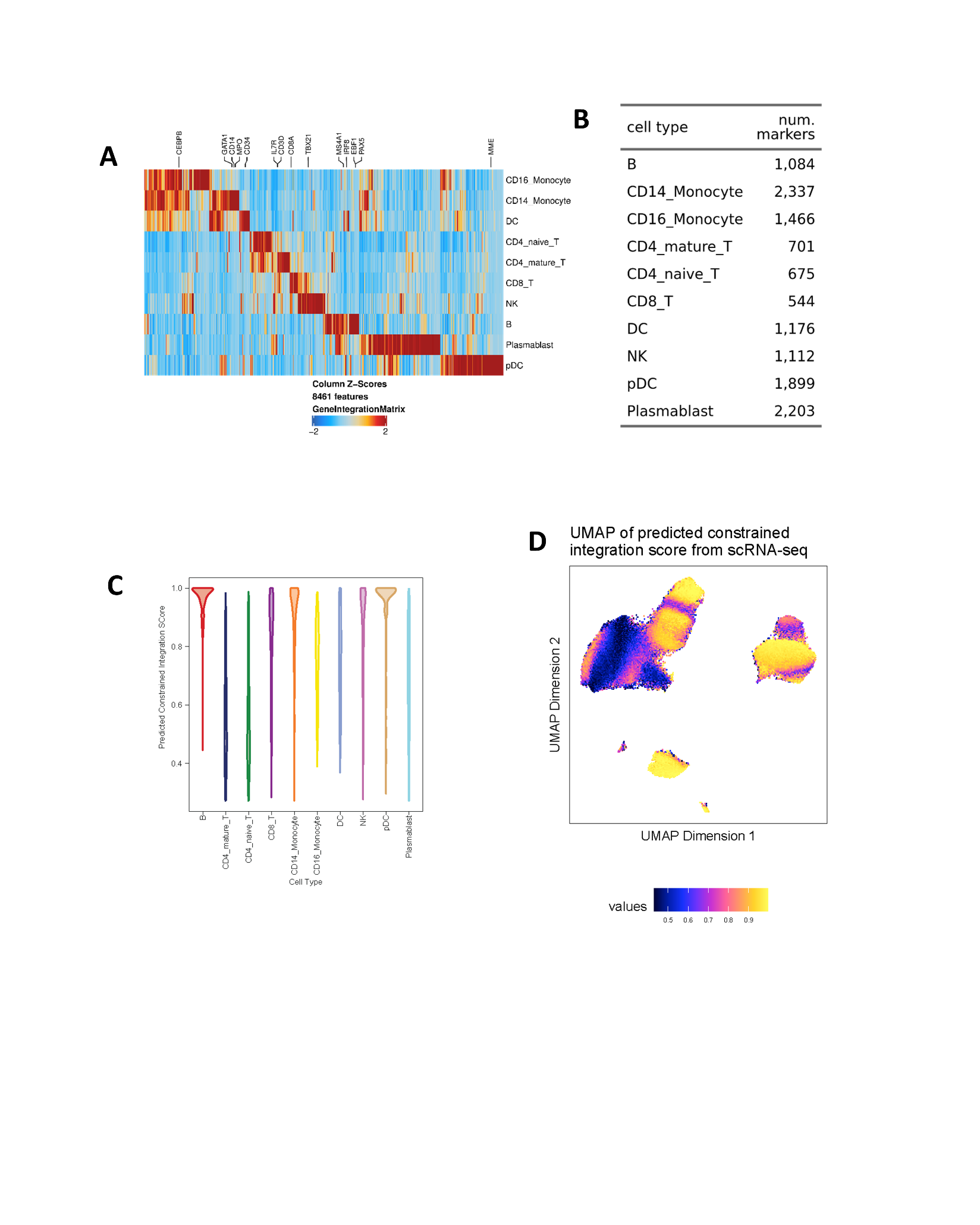
**

**Supplemental Figure S2. (A)** Heatmap of 8461 differentially expressed genes that specifically identify cell subsets in scRNA-seq. **(B)** Table with the number of differentially expressed genes associated with each cell subset. **(C)** Distribution of constrained integration scores for each cell subset between single-cell RNA-seq and ATAC-seq. **(D)** UMAP of scATAC-seq cells labeled by constrained integration scores with scRNA-seq.

**Supplemental Table S1**. Subject serology and PCR results on days when PBMCs were collected for bulk assays.

| **Group** | **Alias ID** | **Race** | **Ethnicity** | **Day of PBMC collection** | | | **PCR** | | | **Serology Test (IgG)** | | |
| --- | --- | --- | --- | --- | --- | --- | --- | --- | --- | --- | --- | --- |
| **Healthy** | 94D44E | White | NR | N/A | | | N/A | | | N/A | | |
|  | 062CD8 | White | NR | N/A | | | N/A | | | N/A | | |
|  | 3F3322 | White | NR | N/A | | | N/A | | | N/A | | |
|  | E0FA1E | White | NR | N/A | | | N/A | | | N/A | | |
|  | 52BA23 | White | NR | N/A | | | N/A | | | N/A | | |
|  | 655A91 | Black | NR | N/A | | | N/A | | | N/A | | |
|  | E9CFE0 | White | NR | N/A | | | N/A | | | N/A | | |
| **Close contacts** | 7768E4 | NR | Non-Hispanic | 0 | 7 | 14 | N | N | N | N | N | N |
|  | 61BBAD | White | Non-Hispanic | 0 | 7 | 14 | N | N | N | N | N | N |
|  | 80A16A | Asian | Non-Hispanic | 0 | 14 |  | N | N |  | N | N |  |
|  | CE0CE8 | White | Non-Hispanic | 0 | 7 | 28 | N | N | N | N | N | N |
|  | 5ABDCB | White | Non-Hispanic | 0 | 14 | 28 | N | N | N | N | N | N |
|  | B96D3B | White | Hispanic | 0 | 14 | 28 | N | N | N | N | N | N |
|  | 6F894A | White | Non-Hispanic | 0 | 7 | 14 | N | N | N | N | N | N |
| **Mild** | 0B943B | White | Non-Hispanic | 0 | 3 | 14 | P | P | P | N | N | P |
|  | 7085CA | White | Non-Hispanic | 0 | 7 | 14 | N^‡^ | N | N | N | N | P |
|  | 2AD75E | Asian | Non-Hispanic | 0 | 7 | 14 | N^‡^ | N | N | N | N | BP |
|  | 450905 | White | Non-Hispanic | 0 | 7 | 14 | P | P | P | N | P | P |
|  | BAAF62 | White | Non-Hispanic | 0 | 7 | 14 | P | P | P | N | P | P |
|  | 1A9B20 | White | Non-Hispanic | 0 | 7 | 14 | P | P | P | N | P | P |
|  | 75A2B6 | Asian | Non-Hispanic | 0 | 7 | 14 | P | P | P | N | P | P |
|  | DF309F | White | Hispanic | 0 | 7 | 14 | N^‡^ | N | N | N | P | P |
| **Moderate** | 180E1A | White | Non-Hispanic | 0 | 7 | 14 | P | N | N | N | P | P |
|  | 82CCF5 | White | Non-Hispanic | 0 | 7 | 14 | P | P | P | N | P | P |
|  | B85D75 | White | Non-Hispanic | 0 | 7 |  | P | P |  | N | P |  |
|  | 40067F | White | Non-Hispanic | 0 | 14 | 21 | P | P | P | N | N | P |
|  | 0BF51C | White | Non-Hispanic | 0 | 7 | 14 | P | P | P | N | P | NR |
|  | 0E1F8E | White | Non-Hispanic | 0 | 7 | 14 | P | P | P | N | P | P |
|  | 3F05F3 | White | Non-Hispanic | 0 | 7 | 28 | P | P | N | N | P | P |

NR, not reported

N, negative; P, positive; BP, borderline positive

^‡^ Positive clinical PCR prior to enrollment, but negative research qPCR

**Supplemental Table S2**. Subject serology and PCR results on days when PBMCs collected for single-cell assays.

| **Group** | **Alias ID** | **Race** | **Ethnicity** | **Day of PBMC collection** | | | **PCR** | | | **Serology Test (IgG)** | | |
| --- | --- | --- | --- | --- | --- | --- | --- | --- | --- | --- | --- | --- |
| **Healthy** | 94D44E | White | NR | N/A | | | N/A | | | N/A | | |
|  | 3F3322 | White | NR | N/A | | | N/A | | | N/A | | |
|  | E0FA1E | White | NR | N/A | | | N/A | | | N/A | | |
|  | 52BA23 | White | NR | N/A | | | N/A | | | N/A | | |
|  | 655A91 | Black | NR | N/A | | | N/A | | | N/A | | |
| **Close contacts** | 7768E4 | NR | Non-Hispanic | 2 | 14 | 28 | N | N | N | N | N | N |
|  | 61BBAD | White | Non-Hispanic | 0 | 7 | 14 | N | N | N | N | N | N |
|  | 80A16A | Asian | Non-Hispanic | 0 | 14 |  | N | N |  | N | N |  |
| **Mild** | 0B943B | White | Non-Hispanic | 0 | 3 | 14 | P | P | P | N | N | P |
|  | 7085CA | White | Non-Hispanic | 0 | 7 | 14 | N^‡^ | N | N | N | N | P |
|  | 2AD75E | Asian | Non-Hispanic | 0 | 7 | 14 | N^‡^ | N | N | N | N | BP |
|  | 450905 | White | Non-Hispanic | 0 | 7 | 14 | P | P | P | N | P | P |
|  | BAAF62 | White | Non-Hispanic | 0 | 7 | 14 | P | P | P | N | P | P |
| **Moderate** | 180E1A | White | Non-Hispanic | 0 | 7 | 14 | P | N | N | N | P | P |
|  | 82CCF5 | White | Non-Hispanic | 0 | 7 | 14 | P | P | P | N | P | P |
|  | B85D75 | White | Non-Hispanic | 0 | 7 |  | P | P |  | N | P |  |
|  | 40067F | White | Non-Hispanic | 0 | 14 | 21 | P | P | P | N | N | P |
|  | 0BF51C | White | Non-Hispanic | 0 | 7 | 14 | P | P | P | N | P | NR |

NR, not reported

N, negative; P, positive; BP, borderline positive

^‡^ Positive clinical PCR prior to enrollment, but negative research qPCR

**Supplemental Methods**

Cohort recruitment and biological sample collection

The **study was approved by** the Duke University Institutional Review Board. Protection of human subjects was in accordance with research protocols approved by the Duke University Institutional Review Board, consistent with the Declaration of Helsinki. Written informed consent was obtained from all research subjects or their legally authorized representatives. Subjects with confirmed or suspected SARS-CoV-2 infection or their close contacts were identified in the outpatient setting and enrolled into the Molecular and Epidemiological Study of Suspected Infection protocol (MESSI, IRB Pro00100241). All close contacts and subjects with mild or moderate COVID-19 were longitudinally sampled from enrollment to convalescent phase. Biological samples were collected prospectively at first visit (Day 0) and at weekly intervals on Day 7 and Day 14. At each visit, infection with SARS-CoV-2 was confirmed using qPCR on nasopharyngeal (NP) swab samples, and serology testing was performed for IgG against the SARS-CoV-2 spike domain (Supplemental Tables 1 and 2). All subjects with mild or moderate COVID-19 progressed from seronegative (IgG-) to seropositive (IgG+). Close contacts were PCR negative and IgG- at all time points; healthy controls were enrolled pre-pandemic and were not tested for SARS-CoV-2 or spike protein IgG. Self-reported symptom surveys were performed at each visit. To categorize symptom severity, the sum of 38 defined symptom categories, each scored 0-4 (0-none, 1-mild, 2-moderate, 3-severe, 4-very severe), was determined from symptom onset through each longitudinal collection.

SARS-CoV-2 IgG ELISA

Antibody response testing was performed using the anti-SARS-CoV-2 spike S1 domain IgG ELISA assay (EUROIMMUN Medizinische Labordiagnostika AG, Lübeck, Germany) according to manufacturer’s instructions. Test results were evaluated by calculating the ratio of the optical density (OD) of the test sample over the OD of the calibrator sample where a ratio of <0.8 was interpreted as negative and ratio of 1.1 or greater as positive; a ratio of 0.8 to <1.1 was considered indeterminate and not used in this study.

SARS-CoV-2 quantification by qRT-PCR

Nasal swab Viral Transport Medium (VTM) was aliquoted and cryopreserved from study subjects to determine SARS-CoV-2 N1 gene copy number by RT-PCR to stratify subjects as COVID PCR positive or negative. Viral RNA was extracted from 140 uL of VTM according to manufacturer’s instructions (QiaAmp Viral RNA minikit). SARS-CoV-2 nucleocapsid (N1) and human RNase P (RPP30) RNA copies were determined using 5 µL of isolated RNA in the CDC-designed kit (CDC-006-00019, Revision: 03, Integrated DNA Technologies 2019-nCoV kit). Standard quantitative RT-PCR (TaqPath 1-step RT qPCR Master Mix, Thermofisher) was run with test RNA and gene-specific standard curves (2e5 copy/mL – 20 copy/mL). Regression analysis was used to determine gene copy number and corrected to report copies/mL of VTM. Samples with a Ct value less than 35 were scored as COVID PCR negative and samples greater than or equal to 35 were scored COVID PCR positive.

Purification of PBMCs

PBMCs were purified using the Ficoll-Hypaque density gradient method according to manufacturer’s instructions. Briefly, whole blood was collected in ACD Vacutainer tubes (BD) and processed within 8 hours by dilution 1:2 in PBS, layered onto the Ficoll-Hypaque (Sigma Aldrich) in 50 ml conical tubes, and centrifuged at 420 x g for 25 minutes. Buffy coat was collected, washed twice in D-PBS by centrifugation at 400 x g for 10 minutes to isolate peripheral blood mononuclear cells (PBMCs) which were assessed for viability and cell count using a Vi-Cell automated cell counter (Beckman-Coulter). PBMCs were adjusted to 10x10^6^ cells/ml in cryopreservation media (90% FBS, 10% DMSO), frozen at -80^o^C using CoolCell LX (BioCision) for 12-24 hours and stored in liquid nitrogen vapor phase.

RNA extraction, total RNA-seq, and data processing

RNA was extracted from 300K cells using the Zymo Direct-zol miniprep kit (Cat# R2051) and RNA quality assessed using the Agilent DNA tape screen assay. The RNA Integrity Number (RIN) scores for all samples were > 7.0. Total RNA libraries were generated using the NuGEN Ovation® SoLo RNA-Seq Library Preparation Kit (Cat# 0500-96). Libraries were sequenced using Illumina NovaSeq 6000 instrument with S4 flow cell and 150 base pair paired-end reads. FASTQ files were generated from the NovaSeq BCL outputs and quality was assessed with FASTQC^1^. Eukaryotic rRNA sequences were removed using SortMeRNA, and the remaining reads were aligned against the hg19 human reference genome using STAR and the following commands: STAR –genomeDir /path/to/STARIndex/ --sjdbGTFfile /path/to/gene.gtf –readFilesIn /path/to/R1.fastq /path/toR2.fastq –runThreadN 8 –twopassMode Basic –outWigType bedGraph –outSAMtype BAM SortedByCoordinate –readFilesCommand zcat –outReadsUnmapped Fastx –outFileNamePrefix $sampleID. Following alignment, the gene count matrix was generated using featureCounts^2,3^.

Differential gene expression analysis

Differentially expressed genes were identified between subjects with different disease severity using the limma package and voom to model variance^4,5^. Subject sex and RNA-seq library batch were added as variables to the design formula to account for expected technical variation in the counts. Genes not annotated as protein coding in the Ensembl hg19 assembly were filtered from the gene count matrix. False discovery rate adjustment was performed for the p-values using the Benjamini-Hochberg procedure. A gene was defined as significantly differentially expressed if the adjusted p-value <= 0.05. Results from limma were passed to the EnhancedVolcano package to generate volcano plots with the same p-value threshold^6^. Two iterations of duplicate correlation and variance modeling implemented in voom were used to estimate the proportion of gene count variance associated with each experimental variable^6^.

Nuclei purification, ATAC-seq and data processing

Nuclei were extracted from frozen PBMCs. Briefly, 100K cells were spun down at 300 x g for 5 minutes at 4^o^C. The supernatant was removed, and cells were mixed with 100 uL of lysis buffer (10mM NaCl, 3mM MgCl2, 10mM Tris-HCl pH7.4, 0.1% Tween-20, 0.1% Nonidet^TM^ P40) and lysed on ice for 4 minutes. Wash buffer (1 mL; 10mM NaCl, 3mM MgCl2, 10mM Tris-HCl pH7.4, 0.1% Tween20) was added before centrifuging at 500 x g for 5 minutes at 4^o^C. ATAC-seq libraries were generated as presented earlier^7^. Briefly, transposition mix (25 μL 2× TD buffer, 2.5 μL transposase (Tn5, 100 nM final), 22.5 μL water) (Illumina Cat# 20031198) was added to the nuclear pellets, incubated at 37 °C for 30 minutes, and samples purified using the Qiagen MinElute PCR Purification Kit (Qiagen Cat#28004). DNA fragments were PCR amplified for a total of 10-11 cycles and resulting libraries purified using the Qiagen MinElute PCR Purification Kit. The libraries were sequenced with an Illumina Novaseq 6000 S4 flow cell using 100 bp paired-end reads. FASTQ files were generated from the NovaSeq BCL outputs and used as input to the ENCODE ATAC-seq pipeline (<https://github.com/ENCODE-DCC/atac-seq-pipeline>) using the MACS2 peak-caller with all default parameters. Output narrowPeak files and aligned BAM files were used for downstream analysis.

Differential chromatin accessibility analysis

Differential accessibility was calculated between groups of subjects with different disease severity using the csaw package^8^. The aligned BAM files were used as input for analysis performed with csaw. First, aligned reads were counted in genome-wide windows of 10 bp, filtering out windows with fewer than 30 reads. Windows were then extended by 2 kb on either size of the window center, and these regions were used to calculate coverage in window flanking regions. Windows with less than 3 fold-change local enrichment were filtered out and not used for statistical modeling. Non-linear normalization was performed with the normOffsets() function. Differential testing was then applied to the window counts using a quali-likelihood negative binomial generalized log-linear model for the relevant experimental contrasts. Differentially accessible windows were defined as having a p-value <= 0.05. Windows were split into three groups: differentially accessible windows (1) only observed in mild subjects; (2) only observed in moderate subjects; and (3) observed in both mild and moderate subjects. These groups of windows were annotated to genomic features using ChIPseeker and used as input to gprofiler2 to perform functional enrichment analysis via the g:Profiler web tool^9^. Gene lists were used to identify enrichment in the TRANSFAC (TF) database, REACTOME (REAC) database, and the molecular function, biological processes, and cellular component gene ontologies (GO: MF, GO: BP, GO: CC).

Single-cell (sc)RNA-seq and data processing

Frozen PBMCs were thawed, and count and cell viability were measured by Countess II. The cell viability exceeded 80% for all samples except PBMC samples from CC subjects, which had viability between 70-80%. For single cell (sc) RNA-seq, 200K cells were aliquoted, spun down, resuspended in 30 uL PBS+0.04%BSA+0.2U/uL RNase inhibitor, and counted using Countess II. GEM generation, post GEMRT cleanup, cDNA amplification, and library construction were performed following 10X Genomics Single Cell 5’ v1 chemistry and quality was assessed using Agilent DNA tape screen assay. Libraries were then pooled and sequenced using Illumina NovaSeq 6000 platform with the goal of reaching saturation or 20,000 unique reads per cell on average. Sequencing data were used as input to the 10x Genomics Cell Ranger pipeline to demultiplex BCL files, generate FASTQs, and generate feature counts for each library. For dimensionality reduction and cell type annotation, gene-barcode matrices generated using CellRanger count were analyzed using Seurat 3 with the default parameters unless otherwise specified^10^. Cells with > 5% of reads mapping to the mitochondrial genome or > 2500 genes detected were removed from the analysis. Counts were log-normalized, and the top 2000 variable features were identified. Principal component analysis was performed using these variable genes, and the top 20 principal components were used for downstream analysis. UMAP dimensionality reduction was performed using the top 20 principal components identified using the Harmony package^11^. Graph-based clustering was performed with resolution = 0.5. Cell types were inferred by using the DatabaseImmuneCellExpressionData() function from the SingleR package^12^. Labels were confirmed by identification of differentially expressed genes using the FindAllMarkers() function from Seurat^10^. For regulatory network inference, the scRNA-seq Seurat object was converted into a SingleCellExperiment and used as input to analysis with the SCENIC package^13^. Cells from seronegative mild and moderate subjects were re-clustered using Monocle 3, and the top 100 marker genes were computed for each cell partition^14^. The standard workflow for running the SCENIC analysis was then performed using the count matrix for these marker genes as input (<https://github.com/aertslab/SCENIC>). Briefly, GENIE3 was used to identify regulons of transcription factors and their downstream regulatory targets with correlated co-expression, and AUCell was then used to score the activity of these regulons in each cluster. The ‘top10perTarget’ co-expression parameter value was used to prune the list of scored regulons. Gene set and pathway enrichment was measured by using the Seurat method AddModuleScore() for lists of genes retrieved from the Molecular Signatures Database v7.5.1 (<https://www.gsea-msigdb.org/gsea/msigdb/>) as documented in Supplemental Table 11. scRNA-seq clusters were merged as follows: Mild/Mod. 1 = clusters 9, 10; Mild/Mod. 2 = cluster 8; Mild/Mod. 3 = cluster 11; Mild/Mod. 4 = clusters 1, 3, 5, 6. The average pathway score was plotted on a heatmap for each cell subset, and hierarchical clustering was applied to group similar samples within each dataset. One-way analysis of variance (AOV) and Tukey testing was performed comparing the average pathway score for each cell subset. The AOV adjusted p-value and the percentage of cell subset contrasts that were significantly associated with the average pathway score were recorded in Supplemental Table 12.

Single-cell (sc)ATAC-seq, data processing and analysis

PBMCs were thawed and nuclei were extracted as for ATAC-seq. The single-cell suspensions of scATAC-seq samples were converted to barcoded scATAC-seq libraries using the Chromium Single Cell 5′ Library, Gel Bead and Multiplex Kit, and Chip Kit (10x Genomics). The Chromium Single Cell 5′ v2 Reagent (10x Genomics, 120237) kit was used to prepare single-cell ATAC libraries according to the manufacturer’s instructions. Quality was assessed using Agilent DNA tape screen assay. Libraries were then pooled and sequenced using Illumina NovaSeq platform with the goal of reaching saturation or 25,000 unique reads per nuclei on average. Sequencing data were used as input to the 10x Genomics Cell Ranger ATAC pipeline to demultiplex BCL files, generate FASTQs, and generate feature counts for each library.

For scRNA-seq and scATAC-seq integration, fragment file outputs generated using CellRanger ATAC count were analyzed using ArchR following the standard workflow and with default parameters unless otherwise specified^15^. Cells with a transcription start site enrichment score < 4, cells with fewer than 1000 detected fragments, and putative doublets were removed from downstream analysis. Dimensionality reduction was computed using iterative latent semantic indexing (LSI), and batch effect correction was applied using Harmony. Graph-based clustering was performed using the FindClusters() method from Seurat 3 with resolution = 0.8. UMAP embeddings were calculated with the top 30 principal components from either LSI or Harmony. Constrained integration was performed using the addGeneIntegrationMatrix() method and scRNA-seq cell type annotations were used to label the identify of scATAC-seq clusters. Pseudo-scRNA-seq gene expression profiles were estimated for each cell type using the addGeneIntegrationMatrix() function from ArchR during the constrained label transfer procedure.

For feature and motif enrichment analysis, peak calling was performed using MACS2 via the addReproduciblePeakSet() method in ArchR which uses pseudo-bulk replicates of cells grouped on a specific design variable. Differentially accessible peaks were identified between two groups and visualized using the ArchR methods getMarkerFeatures() and markerPlot(), respectively. Significance was defined as FDR <= 0.1 and absolute log2 fold change >= 0.5 unless otherwise specified. The ‘cisbp’ motif set was imported from TFBSTools using the ArchR addMotifAnnotations() method, and motif enrichment in differentially accessible peaks was performed using the peakAnnoEnrichment() method. Accessibility estimates were compared to the average gene expression over all cells for the genes that encode each transcription factor. The gene integration matrix was constructed using the .getGroupMatrix() function from ArchR and specifying which subject cohorts to compare.

For integrative analysis with scRNA-seq, the correlations between chromVAR transcription factor deviation scores and gene expression data were calculated using the ArchR method correlateMatrices() to identify activators and repressors. Peak-to-gene linkages were calculated using the addPeak2GeneLinks() method in ArchR using a correlation cutoff of 0.5 and resolution = 1. This approach uses low-overlapping cell aggregates to reduce noise that arises from doing correlative analyses with sparse scATAC-seq datasets. Peak-to-gene linkages were plotted against peak accessibility at gene bodies within DORCs for each cell type. DORC genes were defined as gene loci with > 10 peak-to-gene linkages, and these sites were used as input to the web tool Seanalysis to identify regulation by a known super-enhancer in peripheral blood cells^16,17^. The activity of the top-ranked transcription factor regulators that were correlated with scRNA-seq clusters was estimated for each scATAC-seq cluster by measuring the average genome-wide motif accessibility. These activities were used to calculate Pearson correlation coefficients between scATAC-seq clusters (C1-C8) and scRNA-seq clusters (C1-C11) were calculated to identify scATAC-seq clusters with similar regulatory network activity.

**References**

1 Andrews, S. *FastQC: A Quality Control Tool for High Throughput Sequence Data [Online]*, <<https://www.bioinformatics.babraham.ac.uk/projects/fastqc/>> (2015).

2 Dobin, A. *et al.* STAR: ultrafast universal RNA-seq aligner. *Bioinformatics* **29**, 15-21, doi:10.1093/bioinformatics/bts635 (2013).

3 Kopylova, E., Noé, L. & Touzet, H. SortMeRNA: fast and accurate filtering of ribosomal RNAs in metatranscriptomic data. *Bioinformatics* **28**, 3211-3217, doi:10.1093/bioinformatics/bts611 (2012).

4 Ritchie, M. E. *et al.* limma powers differential expression analyses for RNA-sequencing and microarray studies. *Nucleic Acids Res* **43**, e47, doi:10.1093/nar/gkv007 (2015).

5 Law, C. W., Chen, Y., Shi, W. & Smyth, G. K. voom: Precision weights unlock linear model analysis tools for RNA-seq read counts. *Genome Biol* **15**, R29, doi:10.1186/gb-2014-15-2-r29 (2014).

6 EnhancedVolcano: Publication-ready volcano plots with enhanced colouring and labeling. (R Package, 2021).

7 Buenrostro, J. D., Wu, B., Chang, H. Y. & Greenleaf, W. J. ATAC-seq: A Method for Assaying Chromatin Accessibility Genome-Wide. *Curr Protoc Mol Biol* **109**, 21 29 21-21 29 29, doi:10.1002/0471142727.mb2129s109 (2015).

8 Lun, A. T. & Smyth, G. K. csaw: a Bioconductor package for differential binding analysis of ChIP-seq data using sliding windows. *Nucleic Acids Res* **44**, e45, doi:10.1093/nar/gkv1191 (2016).

9 Raudvere, U. *et al.* g:Profiler: a web server for functional enrichment analysis and conversions of gene lists (2019 update). *Nucleic Acids Research* **47**, W191-W198, doi:10.1093/nar/gkz369 (2019).

10 Stuart, T. *et al.* Comprehensive Integration of Single-Cell Data. *Cell* **177**, 1888-1902.e1821, doi:10.1016/j.cell.2019.05.031 (2019).

11 Korsunsky, I. *et al.* Fast, sensitive and accurate integration of single-cell data with Harmony. *Nature Methods* **16**, 1289-1296, doi:10.1038/s41592-019-0619-0 (2019).

12 Aran, D. *et al.* Reference-based analysis of lung single-cell sequencing reveals a transitional profibrotic macrophage. *Nature Immunology* **20**, 163-172, doi:10.1038/s41590-018-0276-y (2019).

13 Aibar, S. *et al.* SCENIC: single-cell regulatory network inference and clustering. *Nature Methods* **14**, 1083-1086, doi:10.1038/nmeth.4463 (2017).

14 Trapnell, C. *et al.* The dynamics and regulators of cell fate decisions are revealed by pseudotemporal ordering of single cells. *Nature Biotechnology* **32**, 381-386, doi:10.1038/nbt.2859 (2014).

15 Granja, J. M. *et al.* ArchR is a scalable software package for integrative single-cell chromatin accessibility analysis. *Nat Genet* **53**, 403-411, doi:10.1038/s41588-021-00790-6 (2021).

16 Ma, S. *et al.* Chromatin Potential Identified by Shared Single-Cell Profiling of RNA and Chromatin. *Cell* **183**, 1103-1116 e1120, doi:10.1016/j.cell.2020.09.056 (2020).

17 Qian, F.-C. *et al.* SEanalysis: a web tool for super-enhancer associated regulatory analysis. *Nucleic Acids Research* **47**, W248-W255, doi:10.1093/nar/gkz302 (2019).

18 Bravo Gonzalez-Blas, C. *et al.* cisTopic: cis-regulatory topic modeling on single-cell ATAC-seq data. *Nat Methods* **16**, 397-400, doi:10.1038/s41592-019-0367-1 (2019).
